# Facile synthesis of TiO_2_–carbon composite doped nitrogen for efficient photodegradation of noxious methylene blue dye

**DOI:** 10.1039/d4ra05444j

**Published:** 2024-10-28

**Authors:** Victor Onwubiko, Yoshihisa Matsushita, Emad A. Elshehy, Mohamed E. El-Khouly

**Affiliations:** a Nanoscience Program, Institute of Basic and Applied Sciences, Egypt-Japan University of Science and Technology (E-JUST) New Borg El-Arab City Alexandria Egypt mohamed.elkhouly@ejust.edu.eg; b Nuclear Materials Authority El Maasi 11381 Cairo Egypt eelshehy@yahoo.com

## Abstract

The present work shows that the degrading ability of TiO_2_ is significantly improved when exposed to light, particularly in relation to the organic dye methylene blue (MB), following the introduction of a carbon framework through sol-hydrothermal synthesis approach. The newly prepared TiO_2_–C@N composite had the ability to function as both an adsorbent and a photocatalyst to eliminate MB from contaminated wastewater. The outcomes show the removal efficiency of MB amounts to 99.87% upon the application of UV radiation, which is much higher than the rate achieved under dark conditions (28.9%). As ascertained by the kinetic study, the degradation of methylene blue (MB) under UV light through photocatalysis using the TiO_2_–C@N photocatalyst conformed to the widely recognized pseudo-first order (PFO) model. TiO_2_–C@N photocatalyst showed outstanding reliability and reusability, maintaining consistent degradation efficiency over five consecutive cycles without any obvious decline. The materials were characterized by XRD, XPS, FE-SEM, EDS, and N_2_ adsorption–desorption measurements. Nanometer-sized particles, a unique surface dominance, high surface area, large pore volume ratios, low band gap, high oxygen vacancies, increased pollution absorptivity, and reduced electron–hole pair recombination characterize the monolithic TiO_2_–C@N photocatalyst over TiO_2_. These unique features render TiO_2_–C@N a promising catalyst in effectively breaking down noxious MB organic pollutants through photodegradation.

## Abstract 2

The present work demonstrates that the introduction of a nitrogen-doped carbon framework, synthesized through a sol-hydrothermal method, significantly enhances the photocatalytic degradation behavior of TiO_2_ towards methylene blue (MB).

## Graphical abstract

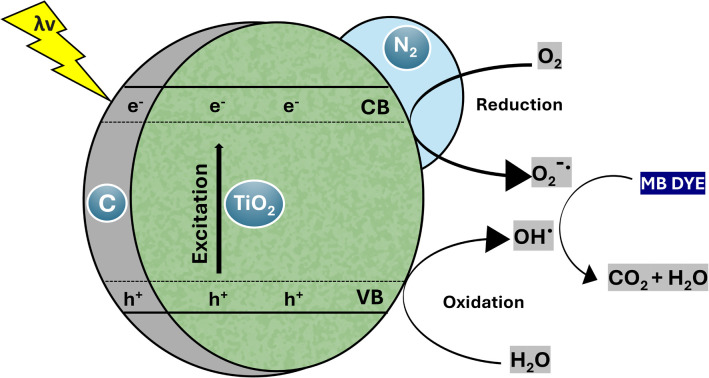

## Introduction

A tremendous problem facing human civilization is the lack of freshwater sources. In the last few decades, emerging pollutants have received great attention since they may have an impact on both public health and environmental quality.^1^ The serious issues arising from the negative impact of these compounds are increasing continually.^2^ Generally, these compounds include pharmaceuticals, hormones and steroids, disinfection by-products, flame retardants, surfactants, agrochemicals, industrial compounds, microplastics, and dyes.^3,4^ The long-term chemical and physical stability of these compounds makes them unable to be decomposed or degraded by conventional treatment processes.^5^ Dyes are considered as common dangerous organic pollutants due to the recent rapid growth of industrial processes.^6^ It has been reported that one of the largest consumers of water is the industry of textiles; however, approximately 15% of all dyes used in the dyeing process are lost to the effluent. Once released, the dye-colored effluent may have a severe impact on the environment, preventing photosynthesis and damaging aquatic life, for example. These aromatic dyes' natural breakdown products tend to bioaccumulate, harming people's health.^7,8^ As a result, creating novel and efficient techniques to purify water free of artificial coloring is essential.

Various methods and materials (physical, chemical, biological) have been identified over time for dye removal from municipal and industrial wastewater.^4,9–12^ Besides the adsorption processes, destructive processes, such as improved oxidation processes and heterogeneous photocatalysis, are also of great interest. The technology of photocatalytic oxidation has been known as the most acceptable approach to treat wastewater due to its high efficacy and lack of secondary pollutants.^13^ As a semiconductor, titanium dioxide (TiO_2_) has gained a favorable reputation over the years due to its extensive application in the degradation of various pollutants found in both air and water. Its remarkable efficiency, particularly in the presence of light, is particularly noteworthy. These materials have many advantages like strong oxidation ability, low cost, abundance, high physical and chemical inertness, with great photostability.^14–16^ The TiO_2_ photocatalytic activity is greatly influenced by various structure-related characteristics.^17^ These properties include phase type,^18^ surface hydrophilicity,^19^ particle size,^20^ crystallinity,^21^ oxygen vacancies,^22,23^ and morphology.^24^ However, TiO_2_ still suffers from some limitations like its large band gap (anatase phase has 3.20 eV and rutile phase has 3.02 eV), poor capacity for pollutant removal, and low quantum efficiency, which result in poor performance in photocatalytic processes. All these limitations restrict the applicability of TiO_2_ alone as photocatalyst in wastewater.^25,26^ Therefore, large number of efforts has been devoted to preparing TiO_2_ composites or TiO_2_ hybridized-materials to address these issues, such as the doping with metals or nonmetals,^27–29^ co-catalysts,^30^ coupling with suitable semiconductors,^31^ and combination with carbon quantum dots (CQDs),^32^ carbon nanotubes (CNTs),^33^ and graphite oxide (GO).^34,35^ Comparable with the pristine TiO_2_, all the prepared composite TiO_2_ based photocatalysts displayed remarkable enhancements in their ability to eliminate the targeted pollutants.

A major improvement is revealed in the composite of titania and carbon. The addition of carbon into TiO_2_ materials enables the tailoring of different carbon compounds to fulfill the specific photocatalytic requirements of TiO_2_, leading to the development of a diverse range of exceptionally efficient photocatalysts. Additionally, carbon materials' high conductivity enables efficient separation of charge carriers, particularly photoexcited free electrons.^32^ Moreover, the carbon-based materials display high surface areas, inert and suitable pore structure, which is critical for enhancing the adsorption capacity toward the targeted pollutants.^36^ In addition to these characteristics, carbon materials possess the desirable qualities of being lightweight, nonreactive, nonpolar, non-toxic, and easily separable from bulk water. Carbon compounds have properties that greatly enhance their desirability for the aim of wastewater treatment.^17^ The addition of a trace amount of carbon to TiO_2_ materials has been shown to decrease the energy band gap and improve its photocatalytic performance in response to visible light. Several methods were reported for supporting TiO_2_ on carbon-based materials, such as precipitation, chemical vapor deposition (CVD), solvothermal and hydrothermal.^37–40^ The obtained TiO_2_–C hybrids can be grouped under one of these categories: carbon-supported, carbon coated, and carbon doped TiO_2_.

Till now, many research groups have utilized prepared TiO_2_–C based photocatalyst to remove synthetic dyes from wastewater. According to Shao *et al.*,^17^ the sol–gel-synthesized anatase TiO_2_–C hybrid aerogel exhibits approximately four times more photocatalytic activity for MB degradation compared to a pure TiO_2_ photocatalyst. Kumar *et al.*'s research^41^ involved the synthesis of carbon-coated TiO_2_ nanoparticles (C-TiO_2_) and their exposure to various thermal treatments. The high specific surface area of, coupled with its capacity to prevent the clustering of TiO_2_ nanoparticles, led to improved photocatalytic performance in degrading both MB dye and crystal violet (CV). When exposed to visible light, the sample that underwent calcination at 800 °C exhibited superior performance. It achieved degradation percentages of 99.72% (*R*%) for CV and MB at 94.13% (*R*%). The rate constants (*K*_1_) for the MB dye and CV dye were measured at 0.0234 min^−1^ and 0.0509 min^−1^, respectively.

The TiO_2_–C composite doped nitrogen was synthesized using the hydrothermal method and subsequently utilized as an adsorbent and photocatalyst under dark and UV light in the degradation of model dye-methylene blue (MB). The photocatalyst obtained was highly porous and its surface area was relatively large. Furthermore, the TiO_2_–C@N photocatalyst showed heightened photocatalytic performance with UV light irradiation in comparison to its performance in the dark. This study examined parameters that impact the ability of the photocatalyst to remove MB, including pH of the pollutant model, contact time for reaction and initial MB concentration. The isotherm of the photocatalytic system and its kinetic models were evaluated to determine its best fit.

## Materials and methods

### Materials

Ethanol (C_2_H_6_O ≥ 95%), pluronic F108 (C_3_H_3_O[C_2_H_4_O]_*x*_[C_3_H_6_O]_*y*_[C_2_H_4_O]_*z*_C_3_H_3_O_2_), sodium hydroxide (NaOH, purity ≥ 98%, pellets anhydrous), titanium isopropoxide also known as TTIP (Ti[OCH(CH_3_)_2_]_4_, ≥97%), urea, CO((NH_2_)_2_), and hydrochloric acid (HCl, 37%) were procured from Sigma-Aldrich (USA) and utilized in their as-received state without further purification. Concentrated methylene blue (MB; C_16_H_18_ClN_3_S, ≥95%) dye was utilized as a representative pollutant in this research study. Deionized water was employed to dilute the initial stock solution of MB, which had a concentration of 1000 mg L^−1^, to achieve the desired concentrations for the experimental trials. The pH of the dye solution was adjusted by utilizing dilute solutions of NaOH base and HCl acid solutions.

### Synthesis of TiO_2_–C@N composite

Using sol-hydrothermal method, the TiO_2_–C@N composite was prepared to function as both an adsorbent and a photocatalyst in the process of removing of MB solution. In the preparatory procedure of TiO_2_–C@N, 8 mL of TTIP was mixed with F108 solution which had a dosage of 5.80 g/20 mL ethanol. The resulting mixture underwent agitation and was raised to a temperature of 45 °C for duration of 30 min. Then, 0.015 g of urea was added and stirred for another 30 min. The mixture that had resulted was then put into a stainless autoclave of 100 mL with Teflon lining and heated for 24 hours at 170 °C. The precipitate acquired in the experiment underwent a sequential washing procedure involving deionized water/ethanol mixture, and dried for 12 hours at 100 °C. Then, the precipitate was subjected to elevated temperatures in a controlled environment consisting of a composite of hydrogen gas and argon gas. This thermal treatment was carried out at 550 °C for 3 hours. This thermal treatment led to the synthesis of a visually distinctive black substance, referred to as the TiO_2_–C@N photocatalyst.

### Characterization methods

The physicochemical properties of TiO_2_–C@N were examined in this study. The surface chemistry of the photocatalyst was analyzed using X-ray photoelectron spectroscopy (XPS), which utilized a monochromatic Al Kα radiation source with an energy of 1486.6 eV. The crystal structure of the catalyst was analyzed with a D8 Advance, A Bruker, Germany X-ray diffractometer (XRD) at 40 kV and 20 mA using Cu Kα radiation (*λ* = 0.15418 nm). The morphology was studied with field emission scanning electron microscopy (FE-SEM, JSM 7800F, JEOL-Japan), which runs at 15 kV accelerating voltage. The instrument is equipped with an elemental analysis mapping system and an energy dispersive X-ray spectrometer (EDS). The Brunauer–Emmett–Teller (BET) method was used in the measurement of the specific surface areas, while Barrett–Joyner–Halenda (BJH) approach quantified the mesoporous and micropore characteristics. The extent of degradation over time was measured with a Biochrom Libra S-22 UV-Vis spectrophotometer.

### Adsorption and photocatalytic activity

In this research, we employed both photocatalytic and adsorption approaches to eradicate MB from a water-based solution. The photodegradation process of MB was carried out in a quartz reactor equipped whilst stirring. For the experiment, a 100 mL aqueous solution contained 50 mg L^−1^ MB solution was prepared. The MB adsorption was conducted in a black wooden box under dark conditions at 300 rpm, and in ambient conditions for 1 hour prior to the UV light experiments. The UV lamp setup has wavelength of 365 nm and the mixture to be radiated was placed at 10 cm from the light source. 3 mL sample of the dye solution was extracted at specific time ranges and subjected to centrifugation in order to prevent interference from the catalyst during UV analysis. The UV-vis spectrophotometer (PerkinElmer Lambda 650s) was used to analyze the absorbance of the tested pollutant solution, which shows a characteristic absorption peak at 664 nm. The MB dye's stability in the dark was proven by the absence of any perceptible changes when the MB solution was exposed to UV radiation or darkness without a photocatalyst. The MB concentration in the solution varied over time, and the MB degradation percentage was ascertained. Eqn (1) shows the computation method for the degradation (%).1
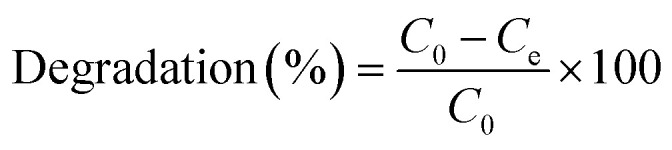


The use of the adsorbent depends on an understanding of the bond between an adsorbent and an adsorbate. In the dark, the same procedure as described earlier for the photocatalytic studies was followed, except that UV lamps were not utilized. A 10 mg dosage of TiO_2_–C@N was introduced into a solution with 100 mg L^−1^ concentrated MB solution of 100 mL. The mixture underwent stirring for a period of 1 hour at room temperature, and the resultant precipitate was isolated from the solution. The residual MB solution was continually monitored using a UV-Vis spectrophotometer, specifically at a wavelength of 664 nm. The adsorption capacity of the TiO_2_–C@N photocatalyst (*q*_e_; mg g^−1^) and the percentage of MB dye removed (*R*%) were quantified using eqn (2) and (3), respectively.^42,43^2
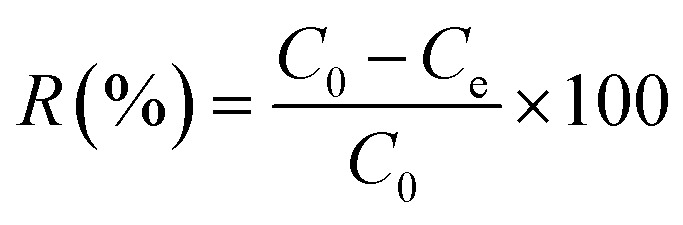
3
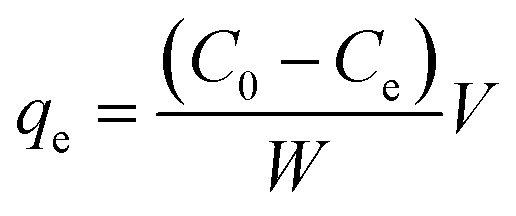


The initial and final MB concentrations, *C*_0_ and *C*_e_, are measured in milligrams per liter, respectively. *V* represents the dye's volume in liters, while *W* represents the adsorbent mass in grams.

## Result and discussion

### Material characterization

#### XRD analysis

**Fig. 1:**
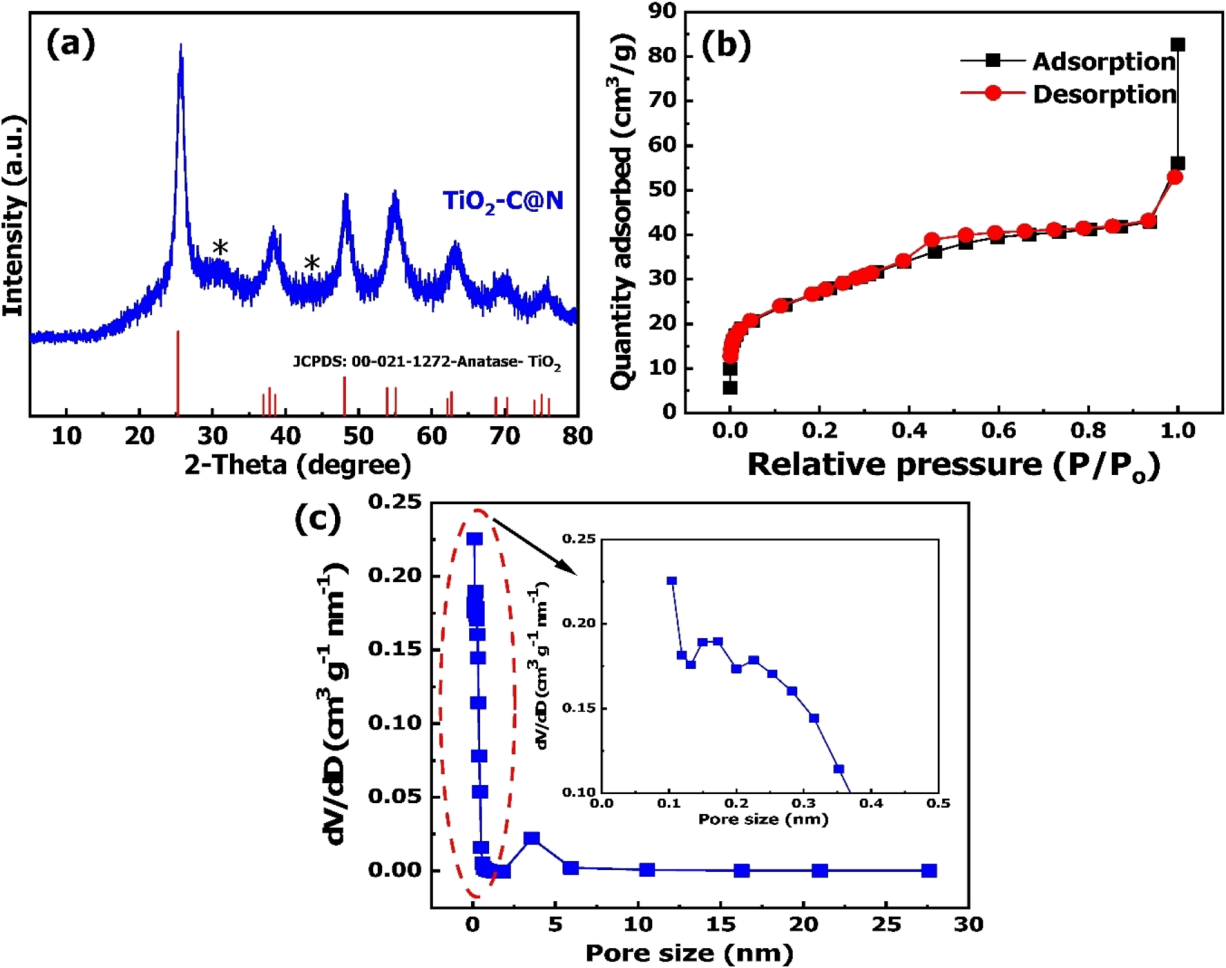
(a) XRD diffractogram, (b) plot of the nitrogen adsorption–desorption isotherm, (c) BJH pore-size distribution curve of the TiO_2_–C@N.

Fig. 1a depicts the XRD diffractogram of TiO_2_–C@N. Its pattern exhibits dominant peaks at 2*θ* values of positions at 25.24°, 37.32°, 47.99°, 53.81°, and 62.54°, correlating to the crystallographic planes of TiO_2_ at positions of (101), (004), (200), (100), and (204), respectively. This corresponds to the anatase phase with the code of JCPDS PDF-21-1272. In addition, its XRD pattern shows modest, diffuse peaks at approximately 25° and 44°, which can be attributed to the crystallographic planes (002) and (100) of the carbon framework.^44^ In case of nitrogen-doped TiO_2_@C, titanium oxide is the only phase present, which shows that these levels of nitrogen-doping do not cause major structural change. The successful synthesis of the TiO_2_–C@N photocatalyst is confirmed.^45–47^ The Debye–Scherrer equation was used to calculate the average size of the TiO_2_–C@N photocatalyst crystallites.^48^ Based on eqn (4), the calculated average crystalline was determined to be 5.15 nm.4
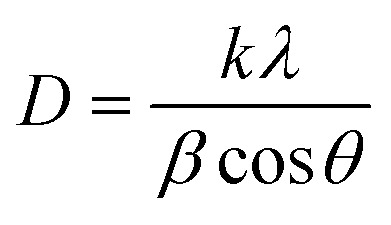
where *D* is the crystallite size in nanometres, *k* (0.94) is the Scherrer constant related to crystallite structure, *λ* is the wavelength of associated radiations, *θ* is Bragg's angle, and *β* is the full width at half-maximum of the diffraction peak.

#### Textural properties

The characteristics of the photocatalytic system are significantly influenced by its specific surface area and pore configuration. To evaluate these characteristics, nitrogen adsorption–desorption isotherms were employed. Fig. 1b depicts the isotherms, showcasing a type of category of IV characteristic and H3-type hysteresis loop. The calculated pore volumes of 0.084 cm^3^ g^−1^ and pore width of 4.47 nm in Fig. 1c validate the mesoporous character of the synthesized materials. This indicates the existence of a mesoporous structure in congruence with the IUPAC group.^46,49,50^ The specific surface area (*S*_BET_) was determined as 95.58 m^2^ g^−1^ using the BET equation, approximately double the value reported for the Bi_2_O_3_/TiO_2_ photocatalyst by Xie *et al.* (42.0 m^2^ g^−1^).^51^

#### SEM and EDS analysis

**Fig. 2:**
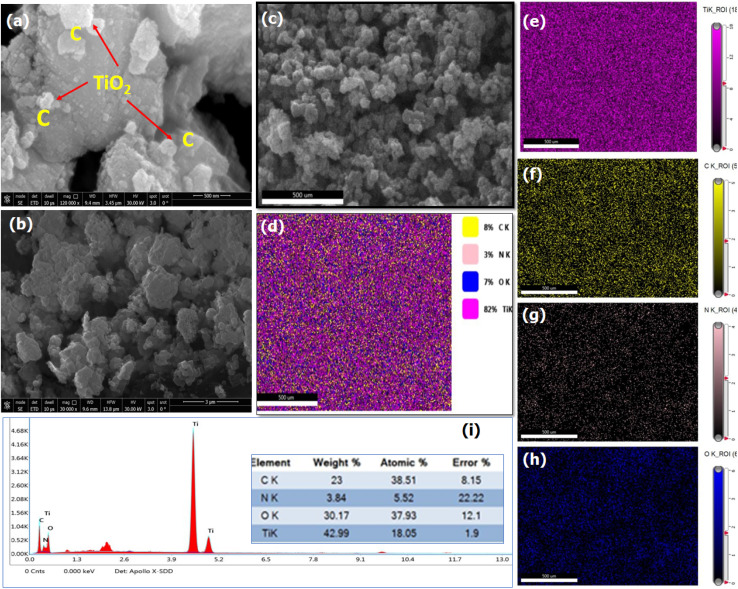
(a–c) FE-SEM images, (d–h) EDS elemental mapping, and (i) spectrum of the EDS of TiO_2_–C@N elemental analysis.

To fully analyze the composition and morphologies of TiO_2_–C@N, SEM and EDS analytical techniques were implemented. The TiO_2_–C@N photocatalyst revealed a variety of particles attached to the carbon framework of varying sizes, as demonstrated by the SEM images in Fig. 2a–c. Furthermore, EDS spectroscopy in Fig. 2d–h confirmed the presence of signals for elemental titanium (Ti), carbon (C), oxygen (O) and nitrogen (N) in TiO_2_–C@N particles. In addition, all presented elements (Ti, O, and C) are distributed homogeneously in the whole EDS analysis area, implying the successful formation of the TiO_2_–C@N photocatalyst. The element distribution along the EDS in Fig. 2i shows that the atomic percentages are 23% (C), 30.17% (O), 42.19% (Ti), and 3.84(N).

#### XPS analysis

**Fig. 3:**
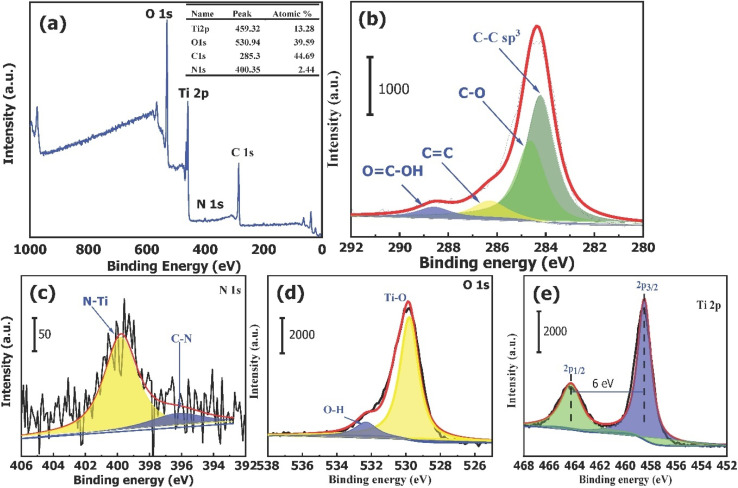
(a) Survey XPS spectrum for TiO_2_–C@N photocatalyst. Spectra for (b) C 1s, (c) N 1s, (d) O 1s, and (e) Ti 2p states.

The XPS survey spectrum of the TiO_2_–C@N photocatalyst (Fig. 3a) shows the existence of titanium, oxygen, carbon, and nitrogen components. The relative proportions of these elements are measured as 13.28%, 39.59%, 44.69%, and 2.44%, respectively. In the high-resolution C1s spectrum (Fig. 3b), binding energies of 283.88, 284.58, 285.48.0, and 288.78 eV had the most prominent peak which are in alignment to C–C, C–O, C=O, and O=C–OH, respectively, as determined through deconvolution analysis.^52^ Additionally, two distinct peaks can be seen in the N1s high-resolution spectra (Fig. 3c). According to the previous study,^53^ the presence of N–Ti bonds can be inferred from the peak observed at 399.98 eV, while the existence of C–N bonds can be suggested from the peak detected at 397.32 eV. Fig. 3d displays the spectra for O1s; two peaks at 529.58 and 531.88 eV are the binding energies. The lower peak in the spectrum can be attributed to the Ti–O bond, whereas the higher peak corresponds to water molecules or hydroxyl groups that have undergone adsorption onto the surface of TiO_2_–C@N. These surface groups enhance the entire photocatalytic performance of the catalyst.^54,55^ The observed energy gap of 70.7 eV between the O(1s) and Ti(2p_3/2_) peaks closely corresponds to the anticipated value of 71.5 eV for TiO_2_, suggesting the successful synthesis of chemically balanced TiO_2_ with no discernible presence of titanium suboxides (TiO_*x*_).^56,57^ The XPS spectra of Ti(2p) are depicted in Fig. 3e. The spectra underwent deconvolution analysis through the application of Voigt curve fitting, in conjunction with the Shirley background subtraction method. The deconvoluted Ti 2p spectrum exhibited a significant doublet at binding energies of 458.48 and 464.68 eV for Ti(2p_3/2_) and Ti(2p_1/2_), respectively, thereby indicating the existence of Ti^4+^ oxidation state within TiO_2_.^58,59^ In addition, the energy difference (Δ*E*) of 2.45 eV observed between the Ti(2p_1/2_) and Ti(2p_3/2_) peaks, along with the corresponding area ratio of 6, provide clear evidence of significant atomic bonding between Ti and O.^59^

### MB adsorption onto TiO_2_–C@N composite

#### Effect of initial pH

**Fig. 4:**
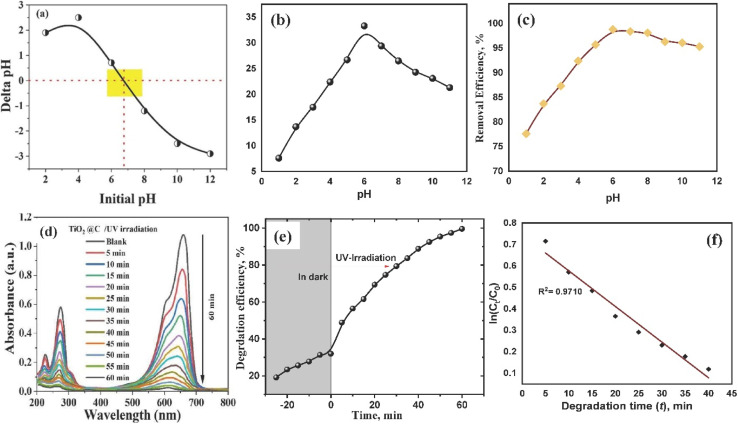
(a) Zeta potential of TiO_2_–C@N photocatalyst in 0.01 M KCl as a function of pH. (b & c) Effect of pH on the removal of MB in the dark and in UV irradiation respectively. (d) Absorption spectra in the degradation process of MB dye in UV light (photodegradation). (e) The impact of duration on the removal efficiency under both dark and UV radiation conditions. (f) First order kinetics for the photocatalytic process.

The point of zero charge (pH_pzc_) of TiO_2_–C@N indicates that the pH value at which the initial and final pH values are equivalent.^60^ It is the pH value at which the surface charge of the particle is effectively zero (Fig. 4a). TiO_2_–C@N exhibited a negative surface charge when the solution pH exceeded 6.75, whereas the charge at the surface was positive when the pH was below 6.75. Previous studies have suggested that anion absorption favors the value of pH lower than the sorbent's pH_pzc_, while cation uptake is preferred at higher pH levels.^61,62^ The sorbent's surface attachment sites and the breakdown process is critical in the removal of dyes, with their effectiveness greatly controlled by the pH level. Consequently, the pH level significantly affects the efficacy of MB removal. To examine the correlation between pH and the percentage of MB removal, the pH values at the initial point were systematically altered within the range of 1 to 11.

Fig. 4b demonstrates the substantial influence of the initial pH level of a 10 mL solution containing 100 mg L^−1^ MB at 25 °C on the removal of the MB by a specified weight of produced material (10 mg) in the dark. Similar conditions were performed under light as seen in Fig. 4c. An investigation was conducted to optimize the performance of TiO_2_–C@N by increasing the initial pH value from 1 to 11. The pH value optimum for MB removal was determined to be 6, which is consistent with recent investigations on MB absorption by fibers from *Posidonia oceanica* (L.).^63^ The pH_pzc_ value of 6.75 for the TiO_2_–C@N photocatalyst provides precise information on the zero-point charge, which is valuable in understanding these findings. Based on the literature, surfaces can acquire a positive charge in instances where the pH is below the point of zero charge (pH_pzc_). This causes an interaction between hydrogen ions (H^+^) and MB cations, leading to a decrease in MB adsorption.^60^ Conversely, at pH levels above the pH_pzc_, the surface gains a negative charge, enhancing the strength of electrostatic forces and attracting MB cations which are positively charged. The cationic character of basic dyes when dissolved in water is the reason for this behavior. As such, the positively charged surface of the adsorbent cannot readily absorb cationic adsorbate species in acidic environments. As the pH of the dye solution rises, a negative charge is induced on the sorbent's surface to improve MB adsorption. This mechanism improves the adsorption process considerably by increasing the electrostatic interaction between the positively charged dye molecules and the negatively charged active sites.^64^ Alhindawy *et al.* reported that raising the solution pH resulted in a higher quantity of groups on the adsorbent surface. This rise in surface negatively charged sites increased the affinity between dye molecules and the adsorbent surface.^61^ Because protons (h^+^) formed on the VB and photo-generated electrons (e^−^) in TiO_2_ moved from the valence band (VB) to the conduction band (CB), the photocatalyst synthesized after irradiation had a higher removal efficiency.^65^ The effectiveness of eliminating the resulting material under both alkalinic and acidic conditions varied considerably. At pH values of 6 and above, the removal efficiency is higher. This suggests that a large concentration of H^+^ ions absorbed on the substance's surface resulted in positive surface charges. The mobility of electrons (e^−^) generated by light is augmented by the existence of positively charged surfaces. The oxygen (O_2_) molecules that are adhered to the surfaces are then impacted by these electrons, creating ˙O_2_^−^ free radicals. Moreover, positively charged surfaces prevent electrons from recombining with h^+^ holes, which increases the amount of OH˙ free radicals produced by the interaction of water with h^+^ sites. The enhanced breakdown of methylene blue (MB) can be attributed to these radical ions OH˙ and ˙O_2_^−^.^43^

In alkaline environments, a comparable phenomenon took place, wherein surfaces possessing negative charges facilitated the migration of holes towards the surface of titania, leading to the generation of the OH˙ radical. The photocatalyst's surface charge and the MB ions' electrostatic interaction hence accelerated the breakdown of MB.^66,67^ Raising the pH of the solution increased the number of functional groups present and, thus, the number of negatively charged sites. As a result, MB molecules were more attracted to the surface they were adsorbed onto.^68^

While protons (h^+^) remain in the valence band, electrons (e^−^) generated by light in TiO_2_ go from it to the conduction band (CB) during irradiation. This characteristic makes the generated photocatalyst more effective in eliminating pollutants. The synthesized material consistently demonstrated high removal efficiency in various acidic and basic environments. Under acidic conditions (pH < 6), the material's surface exhibited a strong affinity for h^+^ ions, resulting in the accumulation of a large concentration of these ions. The accumulation of charge results in the generation of a positive charge on the surface, thereby enhancing the efficiency of the removal process. Additionally, the diminished recombination of electrons and h^+^ holes, as induced by positive surface charges, augments the formation of OH˙ free radicals through the reaction between water and h^+^ sites. Electrons (e^−^) moved around and interacted with adsorbed oxygen (O_2_) molecules to produce ˙O_2_^−^ free radicals. The presence of these extremely reactive ions, hydroxyl radicals, and superoxide radicals may have had a significant role in the methylene blue (MB)'s hastened disintegration. Similar events take place in alkaline conditions, where H^+^ holes move in the direction of TiO_2_'s surface, causing OH˙ radicals to develop on negatively charged surfaces. The photocatalyst's surface charge, which encourages electrostatic interactions with MB ions, facilitates the breakdown of MB.^67^

#### Effect of contact time

Within the specific experimental conditions of 300 rpm, room temperature, pH of 6, and 100 mg L^−1^ of MB concentration, the kinetics of the removal of MB pollutants were investigated. A dosage of 10 mg of TiO_2_–C@N photocatalyst was used. The experiment was conducted in the dark and exposed to UV radiation for duration of 0–60 minutes. Fig. 4d and e presents evidence that clearly indicates the important impact of contact time on the elimination of MB. The adsorption process occurring in the absence of light is noticed to be minimal after a 60 minute run (Fig. 4d). Moreover, under light, much of the elimination occurred within a 30 minute timeframe, with the elimination rate showing a more gradual increase thereafter (Fig. 4e). Thus, it was determined that a duration of 30 minutes is the most effective in achieving equilibrium. The result shows a progressive decline in the highest level of degradation over time under the influence of UV radiation. Fig. 4d illustrates the effectiveness of MB removal under UV radiation. The removal effectiveness was significantly higher with UV radiation compared to the elimination of MB dye in the dark. Despite the high concentration of 100 mg L^−1^, more than 98.6% of MB was effectively removed from the solution due to radiation effects. In contrast, only 32% of MB was removed in the dark environment. Because the semiconductor solution is being exposed to radiation, oxidation reactions are taking place that led to the predicted result.

The objective of the study was to evaluate the extent of MB removal using the TiO_2_–C@N photocatalyst. To achieve this, the time was systematically varied while continuously monitoring the UV-vis reflectance spectra in two distinct conditions: UV light and darkness. The experimental results were validated by employing linear regression analysis on kinetic models, specifically the pseudo-first order and pseudo-second order systems. The pseudo-first order kinetic model was used to explain how the TiO_2_–C@N photocatalyst removed MB in UV light conditions. The equation can be found in its linear version in eqn (5).

5 ln(*C*_*t*_/*C*_o_) = −*Kt*

The rate constant for a pseudo-first order reaction, denoted by *K*, is expressed in terms of min^−1^. The initial concentration of MB is represented as *C*_o_, while the concentration after exposure to UV radiation is denoted as *C*_*t*_. The value of *K* was determined using linear regression analysis, with the slope obtained from Fig. 4f. The pseudo-first order model utilized in the photocatalytic degradation process under UV irradiation was shown to have coefficient of determination, greater than 0.97 with apparent rate constant of 0.02 min^−1^. This suggests a high degree of agreement between the model and the outcomes of the experiments. The results show that a pseudo-first order kinetic model may adequately explain the degradation of the MB organic dye in water when using the TiO_2_–C@N photocatalyst. Moreover, TiO_2_–C@N performs better as a photocatalyst than it does as an adsorbent in terms of getting rid of MB.

#### Effect of initial concentration

The objective of the study is to examine how different MB concentrations affect the TiO_2_–C@N's ability to remove MB in dark or light. 10 mg TiO_2_–C@N, 10 mL of MB solution, pH of 6, and 300 rpm stirring speed are the optimal settings for the experimental setup. It was observed that under dark conditions, the equilibrium adsorption capacity (*q*_e_) of the MB dye consistently increased with increasing concentrations of the MB. To evaluate the adsorption capacity, two different isotherm models—the Freundlich and Langmuir models—were applied. The Langmuir isotherm model, which assumes that adsorption occurs at uniform sorbent sites, states that intermolecular forces rapidly drop with increasing distance from the sorbent's surface. This concept is mathematically represented by eqn (6):6
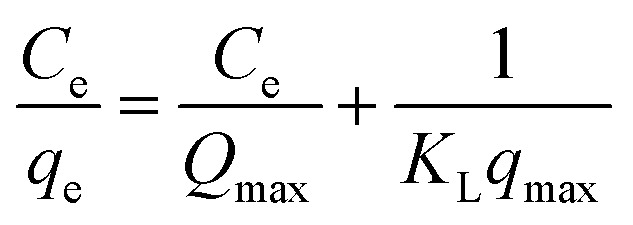


The Langmuir binding constant, or *K*_L_, which is expressed in L mg^−1^ units and indicates the energy required for adsorption, is included in the equation. *C*_e_ is the concentration of MB in the solution at equilibrium (mg L^−1^). The equilibrium adsorption of MB on TiO_2_–C@N is represented by the variable *q*_e_ (mg g^−1^), and the maximum adsorption capacity of TiO_2_–C@N is represented by *Q*_max_ (mg g^−1^). Freundlich isotherm model characterizes the adsorption process as non-ideal and reversible, making it suitable for investigating multilayer adsorption. This model assumes energetic surface heterogeneity.^69–72^ The Freundlich isotherm can be described by two distinct forms: the non-linear model given by eqn (7) and the linear model denoted by eqn (8).

7 *q*_e_ = *k*_F_*c*_e_^1/*n*^ Freundlich-nonlinear model

8
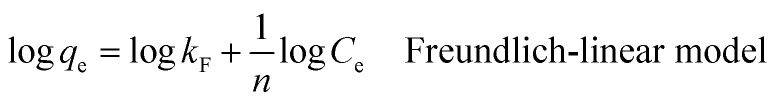


**Fig. 5:**
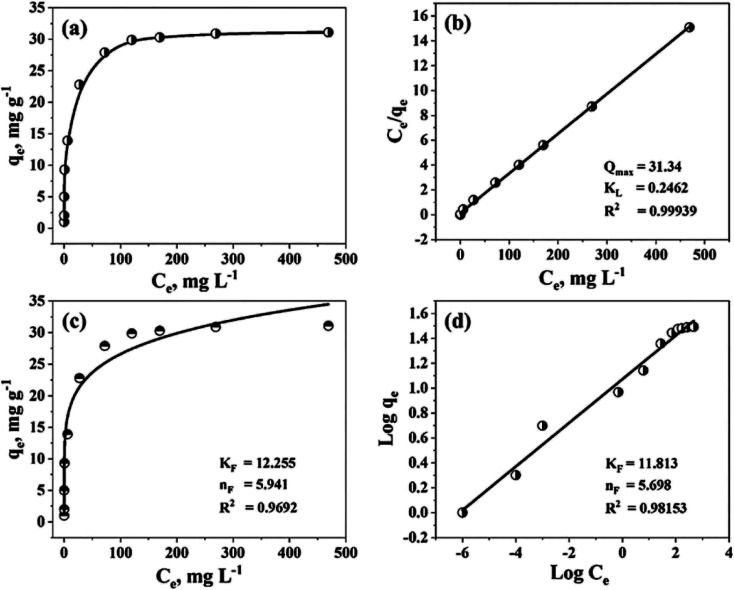
(a) Adsorption isotherm of MB on the TiO_2_–C@N in dark under specific conditions [pH 6, *w* = 10 mg, contact time 30 min], plot of (b) Langmuir, (c) nonlinear Freundlich, and (d) linear Freundlich.

The Freundlich-isotherm nonlinear model is represented by eqn (7), but the Freundlich-linear model is clearly defined by eqn (8). Fig. 5a shows the adsorption isotherms of MB on the composite. The adsorption intensity and capacity are measured by the Freundlich constants, *k*_F_ and *n*, respectively. Fig. 5b depicts the experimental data using Langmuir isotherms, while Fig. 5c and d represents the data using Freundlich isotherms. All relevant parameters are included in Table 1. The resultant *Q*_max_, or Langmuir adsorption capacity, matches the values found in the experiment. The adsorption intensity and capacity are represented by the Freundlich constants, *n* and *k*_F_, respectively. The Langmuir model's better representation of the experimental data over the Freundlich model is further supported by the *R*^2^ values, which are a trustworthy measure of accuracy. Compared to the non-linear least-squares method, the linear method yields higher *R*^2^ values for the Freundlich isotherm parameters. Additionally, Fig. 5c and d shows that compared to the Langmuir models, the Freundlich isotherm models have a greater coefficient of determination. This illustrates the active sites are uniform on the surface of TiO_2_–C@N.

**Table 1:** Langmuir and Freundlich parameters for the adsorption of MB onto TiO_2_–C@N in the dark

*q*_max_ (mg g^−1^)	Langmuir parameters			Nonlinear Freundlich parameters			Linear Freundlich parameters		
	*Q*_max_ (mg g^−1^)	*K*_L_ (L mg^−1^)	*R* ^2^	*k*_F_ (L g^−1^)	*n*	*R* ^2^	*k*_F_ (L g^−1^)	*n*	*R* ^2^
31.1	31.34	0.2462	0.999	12.255	5.941	0.969	11.813	5.698	0.981

**Fig. 6:**
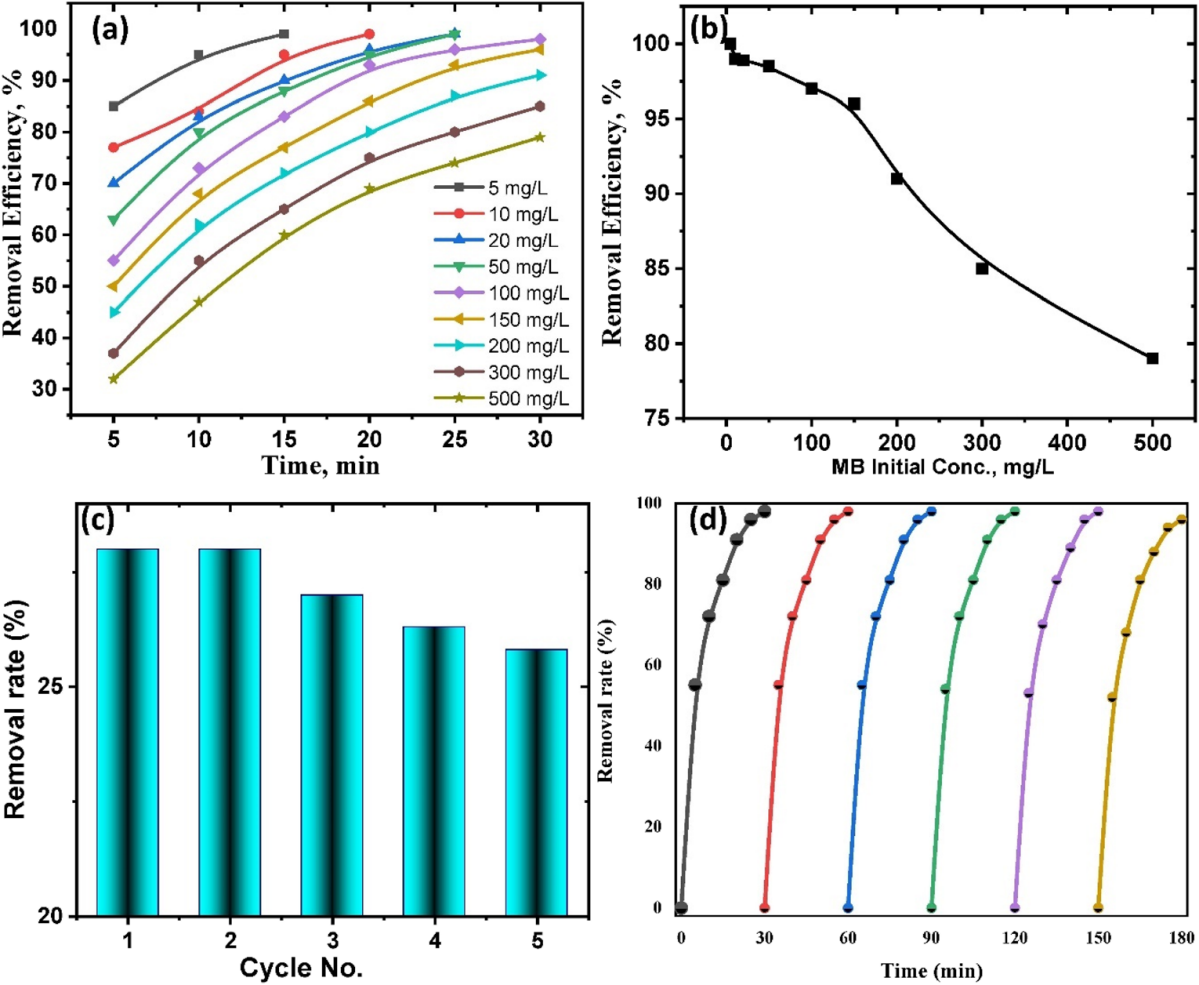
(a & b) Photodegradation efficiency of MB by TiO_2_–C@N as a function of MB initial concentrations at pH 6. Reusability of TiO_2_–C@N photocatalyst in the (c) dark, and (d) UV light.

The initial concentrations of MB have an impact on TiO_2_–C@N photocatalytic performance, which in turn has an impact on MB's susceptibility to deterioration under UV radiation. The degradation of MB by the TiO_2_–C@N photocatalyst is the primary goal of the work. MB's initial concentration varies while maintaining the ideal values of all other parameters. Various concentrations of MB (5, 10, 20, 50, 100, 150, 200, 300, and 500 mg L^−1^) are produced and subjected to UV light testing at different time intervals (Fig. 6a and b). Under optimal testing conditions, the TiO_2_–C@N photocatalyst successfully removes 97.8% of the 100 mg L^−1^ of MB. Fig. 6a indicates that, as the initial concentration of MB with 100 mg L^−1^ surpasses, the degradation percentage decreases. After 30 minutes, the degradation percentage at a concentration of 500 mg L^−1^ is 79%.

The development of ecologically beneficial, viable, and low-cost waste management systems is facilitated by stability over time and reusability in waste management.^73^ The construction of closed-loop systems that can accomplish ecologically benign waste management is made feasible through the reuse of photocatalysts.^74^ Consequently, it is imperative to assess the photocatalyst's recyclability and longevity in these processes. The TiO_2_–C@N photocatalyst can be efficiently eluted using a 1 M HNO_3_ solution. Additionally, the photocatalyst demonstrated the ability to be regenerated and reused for up to five cycles in the adsorption process (Fig. 6c) and up to six cycles in the photocatalytic degradation process (Fig. 6d). These findings indicate a slight decrease in efficiency and that the TiO_2_–C@N photocatalyst exhibits remarkable stability when utilized repeatedly. The impact of the stripping agents used in the recycling methods can be ascribed for the decline in TiO_2_–C@N performance. Due to its exceptional efficacy, reusability, and user-friendliness, the TiO_2_–C@N photocatalyst can effectively eliminate organic pollutants from wastewater.

### Proposed photocatalysis mechanism

**Fig. 7:**
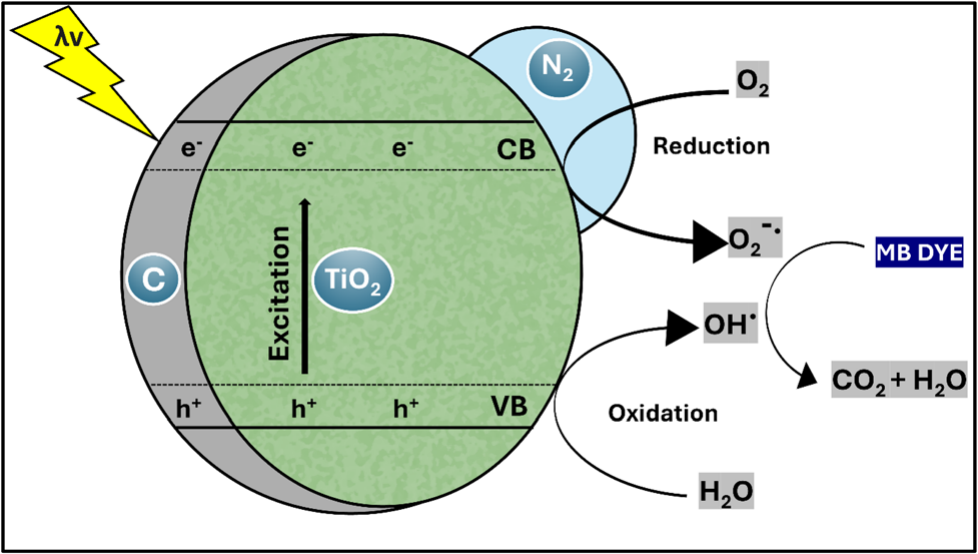
Photocatalytic degradation diagram of TiO_2_–C@N photocatalyst.

The objective of the experiment was to assess the efficiency of TiO_2_–C@N in reacting with UV light for the purpose of undergoing photocatalysis and decomposing methylene blue (MB) in water. Semiconductor materials often exhibit high efficacy in the photocatalytic process, effectively decomposing organic pollutants. Therefore, we examined the efficiency of removing MB by utilizing the nanomaterial generated under two conditions: without exposure to light and under UV light exposure. When exposed to irradiation, the novel hybrid nanocomposite demonstrated much higher removal efficiency as compared to when it was retained in the absence of light. Even in the absence of light treatment, the doping of carbon into the TiO_2_–C@N composite enhanced TiO_2_'s capacity to adsorb MB *via* promoting π–π interactions between MB and carbon. Improved surface-to-liquid medium interaction was facilitated by the TiO_2_–C@N composite's larger surface area. The enhanced removal efficacy observed under UV irradiation can be attributed to oxidation processes resulting from the photocatalytic mechanism (Fig. 7). The material subjected to investigation exhibited suppressed electronic recombination and enhanced mass transfer, leading to a significant acceleration of the removal process within a short timeframe. The introduction of carbon during the synthesis process reduces the energy gap between the valence and conduction bands.^75,76^ This broadens the spectrum of light absorption and facilitates the efficient transfer of electrons from titanium oxide to the carbon sheets. As a result, recombination is effectively reduced.

## Conclusion

This study employed a sol-hydrothermal technique to fabricate a mesoporous TiO_2_–C@N photocatalyst. The photocatalyst was subsequently employed to degrade methylene blue solution. Using BET, XRD, XPS, and SEM-EDS methods, the structure and morphology of the TiO_2_–C@N photocatalyst were assessed. The application of UV radiation resulted in a significant degradation efficiency of 99.87% for MB, which was much higher compared to the 28.9% achieved in the dark. Under UV light, it was observed that the degradation of MB followed a pseudo-first-order model when utilizing the TiO_2_–C@N photocatalyst. This finding implies that the utilization of TiO_2_–C@N as a photocatalyst, as opposed to an adsorbent, enhances its efficacy in the removal of MB. Furthermore, the TiO_2_–C@N photocatalyst exhibited remarkable durability and reusability over five consecutive cycles, maintaining consistent degradation efficiency without any noticeable decline. In conclusion, this study presents new possibilities for creating highly efficient and economical photocatalysts using TiO_2_ for photodegradation toxic organic dyes and wastewater treatments.

## Data availability

Data will be made available on request.

## Conflicts of interest

The authors declare no conflict of interest.
